# Natriuretic Peptide and Transthyretin and Their Relationship With Clinical Outcomes: Results From ATTRIBUTE‐CM, a Randomized Controlled Trial

**DOI:** 10.1161/JAHA.126.050566

**Published:** 2026-08-30

**Authors:** Onyedika John Ilonze, Daniel Philip Judge, Ahmad Masri, Prem Soman, Francesco Cappelli, Michel Georges Khouri, Nitasha Sarswat, Mazen Hanna, Yuxi Liu, Gillian Murtagh, Kuangnan Xiong, Adam Castaño, Jean‐François Tamby, Jonathan C. Fox, Uma Sinha, Jan Marie Griffin, Julian D. Gillmore, James Louis Januzzi

**Affiliations:** ^1^ Division of Cardiovascular Medicine, Krannert Cardiovascular Research Center Indiana University School of Medicine Indianapolis IN USA; ^2^ Division of Cardiology Medical University of South Carolina Charleston SC USA; ^3^ Division of Cardiology Oregon Health and Science University Portland OR USA; ^4^ Division of Cardiology University of Pittsburgh Medical Center Pittsburgh PA USA; ^5^ Tuscan Regional Amyloidosis Centre, Department of Clinical and Experimental Medicine Careggi University Hospital Florence Italy; ^6^ Duke University School of Medicine Durham NC USA; ^7^ University of Chicago Medicine Chicago IL USA; ^8^ Cleveland Clinic Cleveland OH USA; ^9^ Cardiology Division, Massachusetts General Hospital Harvard Medical School Boston MA USA; ^10^ BridgeBio Pharma, Inc. San Francisco CA USA; ^11^ National Amyloidosis Centre, Division of Medicine University College London, Royal Free Hospital London UK; ^12^ Baim Institute for Clinical Research Boston MA USA

**Keywords:** acoramidis, ATTR‐CM, ATTRIBUTE‐CM, NT‐proBNP, transthyretin, Heart Failure

## Abstract

**Background:**

Transthyretin amyloid cardiomyopathy (ATTR‐CM) carries high risk for all‐cause mortality (ACM), cardiovascular mortality (CVM), and cardiovascular hospitalization (CVH). NT‐proBNP (N‐terminal pro‐B‐type natriuretic peptide) and serum TTR (sTTR) are prognostic biomarkers in transthyretin amyloid cardiomyopathy, but their combined value for integrated risk stratification remains unknown. We evaluated the relationship between baseline NT‐proBNP and sTTR and their prognostic value in ATTRIBUTE‐CM (Efficacy and Safety of Acoramidis in Transthyretin Amyloid Cardiomyopathy).

**Methods:**

ATTRIBUTE‐CM was a phase 3, randomized, double‐blind, placebo‐controlled trial. Among 632 participants randomized to acoramidis or placebo, baseline NT‐proBNP and sTTR concentrations were evaluated post hoc for associations with baseline characteristics, Kansas City Cardiomyopathy Questionnaire overall scores, 6‐minute walk distance, National Amyloidosis Center stage, Geriatric Nutritional Risk Index score, and subsequent time to ACM/first CVH and CVM/first CVH through Month 30.

**Results:**

Higher baseline NT‐proBNP and lower sTTR were associated with greater disease severity and were inversely correlated. Using multivariable linear regression, sTTR remained inversely correlated with NT‐proBNP. Using multivariable Cox analyses, baseline NT‐proBNP and sTTR were each independently associated with ACM/first CVH and CVM/first CVH. Higher baseline NT‐proBNP combined with lower sTTR conferred higher risk and shorter time to event. Acoramidis reduced risk of ACM/first CVH and CVM/first CVH regardless of baseline NT‐proBNP/sTTR, National Amyloidosis Center stage, or Geriatric Nutritional Risk Index.

**Conclusions:**

Baseline NT‐proBNP and sTTR were additively prognostic for ACM/first CVH and CVM/first CVH in patients with transthyretin amyloid cardiomyopathy. Their joint measurement may be useful for enhanced staging, risk stratification, and prognostication in transthyretin amyloid cardiomyopathy. Acoramidis demonstrated consistent clinical efficacy regardless of baseline NT‐proBNP/sTTR status.

**Registration:**

URL: https://clinicaltrials.gov/study/NCT03860935; Unique Identifier: NCT03860935. Registered: February 2019.

## Graphical abstract

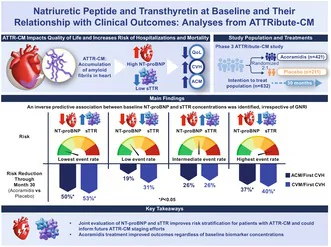

**Nonstandard Abbreviations and Acronyms**
6MWD: 6‐minute walk distance
ATTR‐CM: transthyretin amyloid cardiomyopathy
ATTRIBUTE‐CM: phase 3 pivotal trial of acoramidis in ATTR‐CM (Efficacy and Safety of Acoramidis in Transthyretin Amyloid Cardiomyopathy)
GNRI: Geriatric Nutritional Risk Index
KCCQ: Kansas City Cardiomyopathy Questionnaire
NAC: National Amyloidosis Centre
sTTR: serum transthyretin

**Clinical Perspective**

**What Is New?**

- Baseline NT‐proBNP (N‐terminal pro‐B‐type natriuretic peptide) and serum transthyretin were independently and additively prognostic for all‐cause mortality/cardiovascular hospitalization and cardiovascular mortality/cardiovascular hospitalization in transthyretin amyloid cardiomyopathy.
- Higher NT‐proBNP combined with lower serum transthyretin identified patients at greatest risk, regardless of nutritional status. Acoramidis reduced risk of clinical events irrespective of baseline biomarker concentrations.

**What Are the Clinical Implications?**

- Joint assessment of NT‐proBNP and serum transthyretin may enhance staging and risk stratification in transthyretin amyloid cardiomyopathy beyond traditional clinical measures. Treatment benefit with acoramidis is maintained across biomarker‐defined risk groups. Combined biomarker evaluation may help inform prognosis while supporting treatment decisions in transthyretin amyloid cardiomyopathy.

Transthyretin amyloid cardiomyopathy (ATTR‐CM) is a progressive, systemic, and typically fatal disease if left untreated.^1^, ^2^ ATTR‐CM is characterized by destabilization of transthyretin tetramers, which dissociate into unstable monomers that misfold and aggregate into toxic amyloidogenic oligomers, ultimately leading to the deposition of amyloid fibrils in multiple tissues, including the heart and nerves.^3^, ^4^, ^5^ Progressive accumulation of amyloid fibrils in the myocardium primarily manifests as a restrictive cardiomyopathy, with progressive and life‐threatening heart failure (HF).^3^

At present, accepted prognostic measures in ATTR‐CM include HF‐related health status, functional capacity, diuretic use, and imaging findings.^6^, ^7^, ^8^ Additionally, circulating biomarkers such as creatinine (used to estimate glomerular filtration rate [eGFR]) and NT‐proBNP (N‐terminal pro‐B‐type natriuretic peptide, released in response to myocardial wall stretch and volume overload) provide important prognostic and staging information beyond traditional clinical variables.^9^, ^10^, ^11^, ^12^, ^13^ These biomarkers have been incorporated into established staging systems for ATTR‐CM,^8^, ^12^, ^13^ including the National Amyloidosis Center (NAC) and Mayo Clinic systems, both of which incorporate NT‐proBNP. Despite its role in commonly used staging systems, a more mechanistic understanding of NT‐proBNP in ATTR‐CM is needed, particularly with the emergence of serum transthyretin (sTTR) as a candidate ATTR‐CM biomarker. sTTR has not yet been incorporated into staging systems; therefore, a better understanding of how sTTR and NT‐proBNP are interrelated is needed. This is particularly important with the introduction of TTR stabilizers as a treatment option for the disease.

TTR stabilizers bind to the TTR tetramer, preventing its dissociation into amyloidogenic monomers.^14^ Stabilizers are an effective therapeutic class for the treatment of ATTR‐CM^15^; notably, stabilization of TTR is associated with increases in sTTR.^15^, ^16^, ^17^ In the phase 3 ATTRIBUTE‐CM (Efficacy and Safety of Acoramidis in Transthyretin Amyloid Cardiomyopathy; NCT03860935) study, near‐complete (≥90%) stabilization of TTR with acoramidis was associated with an early and significant rise in sTTR (by Day 28) that was sustained through Month 30.^15^ This early increase in sTTR was associated with improved survival and remained a strong multivariable predictor of outcomes after adjustment for other known predictors such as TTR genotype status, New York Heart Association class and NAC stage.^17^ sTTR concentrations may thus represent a novel ATTR‐CM disease‐specific biomarker that could additively inform disease prognostication in ATTR‐CM, but further evaluation is warranted.^17^

The present analysis examined baseline NT‐proBNP and sTTR data from ATTRIBUTE‐CM participants. We aimed to (1) understand the variance in demographic and clinical characteristics of participants according to their baseline biomarker concentrations; (2) identify baseline factors independently associated with biomarker concentrations; (3) characterize clinical outcomes as a function of baseline biomarkers, both individually and collectively; and (4) evaluate the effect of acoramidis on composite outcomes of all‐cause mortality (ACM), cardiovascular mortality (CVM), and time‐to‐first cardiovascular hospitalization (CVH) as a function of the combined baseline NT‐proBNP and sTTR profile, a novel integrative approach to risk stratification in ATTR‐CM.

## METHODS

### Data Availability Statement

Qualified academic investigators may submit requests for access to data to medinfo@bridgebio.com. Requests for access to study data will be evaluated by BridgeBio Pharma, Inc., and access will be provided contingent on the approval of a research or study proposal and the execution of a data sharing agreement. BridgeBio Pharma, Inc., will consider requests for access to participant‐level data if participant privacy is ensured through methods such as data deidentification, pseudonymization, or anonymization (as required by applicable law) and if such disclosures were included in the informed consent form or study protocol of the relevant study.

### Study Design

Full details of the rationale and study design of ATTRIBUTE‐CM have been previously published.^15^ Briefly, ATTRIBUTE‐CM was a multicenter, phase 3, double‐blind, randomized, placebo‐controlled trial designed to assess the efficacy and safety of acoramidis compared with placebo in patients with ATTR‐CM. Participants were randomly assigned in a 2:1 ratio to receive acoramidis hydrochloride (800 mg) or matching placebo tablets administered orally twice daily for 30 months. Stratification was based on TTR genotype, NT‐proBNP levels, and eGFR levels according to the NAC staging criteria.^12^ Treatment with tafamidis was not permitted during the initial 12 months of the trial, although such treatment was permitted thereafter at the discretion of the investigator.

This study was conducted in accordance with the International Conference on Harmonization Good Clinical Practice guidelines and the principles of the Declaration of Helsinki. All participants provided written informed consent, and the study was approved by an ethics committee at each participating site.

In the ATTRIBUTE‐CM trial, the safety population (intention‐to‐treat) included randomized study participants who had received at least 1 dose of acoramidis or placebo; this population was used for the present analysis, given the goals to understand baseline biomarker implications.

### Selection Criteria

The full inclusion and exclusion criteria and numbers of participants excluded following randomization for the ATTRIBUTE‐CM trial can be found in the report of the primary results.^15^ Briefly, to be eligible for enrollment, participants had to be between the ages of 18 and 90 years and have an established diagnosis of ATTR‐CM. The study population was representative of the broader patient population with ATTR‐CM.

### Variables and Outcomes of Interest

Baseline demographics, including race and sex, and clinical data were collected at the time of enrollment in the trial. The New York Heart Association class and 6‐minute walk distance (6MWD) were used to assess functional status, and the Kansas City Cardiomyopathy Questionnaire (KCCQ) was used to evaluate HF‐related health status; KCCQ scales included the overall summary score, clinical summary score, and total symptom score. Post hoc analyses for composite outcomes (ACM/first CVH and CVM/first CVH) through Month 30 were performed.

### Nutrition

The prognosis of HF syndromes (including ATTR‐CM) may be influenced by nutritional status. Furthermore, sTTR (also known as prealbumin) is known to be associated with nutritional status. To assess the possibility that malnutrition modified the findings in this analysis, we assessed nutrition using a validated and reproducible equation, the Geriatric Nutritional Risk Index (GNRI),^18^ and examined whether nutrition modified results from biomarker testing.

### Biomarkers and National Amyloidosis Center Staging

Concentrations of NT‐proBNP were measured from baseline blood samples using an electrochemiluminescence immunoassay (Elecsys NT‐proBNP; Roche Diagnostics, Rotkreuz, SE), and sTTR was measured using a turbidimetric immunoassay (ARCHITECT TTR; Abbott Diagnostics, Abbott Park, IL, USA). We also aimed to determine whether the combination of NT‐proBNP and sTTR provides incremental benefit to the NAC staging system.^12^ To assess this, we evaluated the change in the C‐statistic of models containing NT‐proBNP/sTTR with and without NAC staging and examined model calibration using the Akaike information criterion (AIC) and Bayesian information criterion (BIC).

### Statistical Analysis

Continuous variables were summarized as mean±SD or median (quartile [Q]1–Q3), as appropriate. Differences in baseline characteristics across biomarker quartiles (NT‐proBNP and sTTR) were compared using the 1‐way ANOVA for normally distributed continuous variables and the Kruskal–Wallis test for nonnormally distributed continuous variables. Categorical variables were analyzed using the chi‐square test or Fisher's exact test when expected cell counts were <5. No imputation was performed; the total number of missing values was 9 (1.4% of all data points). Associations between baseline log2‐transformed NT‐proBNP and log2‐transformed sTTR concentrations were assessed using Spearman correlation. Correlates of baseline biomarker concentrations were evaluated with univariable and multivariable linear regression models. Confounders examined were age, sex, race, TTR genotype, eGFR, New York Heart Association class, and GNRI. Variables for multivariable models were then selected using the least absolute shrinkage and selection operator.

Time‐to‐event analyses were performed using Cox proportional hazards models to estimate hazard ratios (HRs) and 95% CIs for the association of baseline biomarkers with ACM/first CVH and CVM/first CVH outcomes. To align with methodology from the initial ATTRIBUTE‐CM report,^15^ multivariable models were initially adjusted for treatment assignment, baseline 6MWD, and baseline eGFR. Then, individual biomarkers (NT‐proBNP or sTTR) were individually added; subsequent joint models were created with both biomarkers added simultaneously. Discrimination was quantified using Harrell's C‐statistic, with 95% CIs derived from the SE of the concordance estimate. Model parsimony was assessed using the AIC and BIC, where lower values indicate better fit penalized for model complexity. Formal likelihood ratio tests were used to evaluate whether the addition of the second biomarker to a model already containing the first resulted in a statistically significant improvement in model fit. To explore treatment effects across biomarker‐defined subgroups, HRs for acoramidis versus placebo were subsequently estimated in unadjusted Cox models. Interaction testing evaluated for the impact of nutrition on the impact of acoramidis. The proportional hazards assumption was checked using the Grambsch–Therneau test based on scaled Schoenfeld residuals and found to be preserved. For modeling analyses not requiring categorization, continuous log2‐transformed concentrations of sTTR and NT‐proBNP were used. For display purposes, when dichotomization was necessary, biomarker concentrations were expressed as medians. A 2‐sided *P* value <0.05 was considered statistically significant. All analyses were post hoc and not α‐controlled; *P* values are presented without adjustment for multiple comparisons. All analyses were conducted using R software, version 4.5.1 (R Foundation for Statistical Computing, Vienna, Austria).

## RESULTS

### Baseline Characteristics

Overall, the mean age of the study participants was 77.3±6.6 years.^15^ Women constituted 9.8% of the study population.^15^ In terms of race, most of the cohort was White (87.8%), and Black and Asian races represented 4.7% and 2.1% of the participants, respectively.^15^ Most of the participants had wild‐type ATTR‐CM (90.3%).^15^ The median eGFR was 61.0±18.3 mL/min per 1.73 m^2^. The median GNRI (Q1–Q3) for the overall population was 104.24 (101.26–107.22).

NT‐proBNP concentrations were available in all (N=632) study participants in the safety data set at baseline, and sTTR concentrations were available in n=626. Participants had a baseline median (Q1–Q3) NT‐proBNP of 2326 (1282–3898) ng/L. sTTR levels were in the low‐normal range at baseline, with a baseline median (Q1–Q3) sTTR of 23 (20–27) mg/dL (6 participants were missing baseline sTTR data; the core laboratory reference range is 20–40 mg/dL).

Baseline clinical characteristics of the study participants by baseline NT‐proBNP and sTTR quartiles are detailed in Table 1. Across baseline quartiles of NT‐proBNP, an inverse association with median concentrations of sTTR was present; sTTR was more likely to be lower at higher NT‐proBNP concentrations (*P* < 0.001 across NT‐proBNP quartiles; Table 1). Across baseline quartiles of sTTR, an inverse association with median NT‐proBNP concentration was present; NT‐proBNP was more likely to be lower at higher sTTR concentrations (*P*<0.001 across sTTR quartiles; Table 1). Notably, high GNRI (indicating better nutrition) was associated with lower NT‐proBNP and higher sTTR.

**Table 1:** Clinical Characteristics by Quartiles of Baseline NT‐proBNP and Baseline sTTR

	Clinical characteristic by quartiles of baseline NT‐proBNP				*P* value
	Q1 (n=158)	Q2 (n=158)	Q3 (n=158)	Q4 (n=158)	
NT‐proBNP, ng/L, median (Q1–Q3)	827 (556–1021)	1812 (1448–2031)	2872 (2522–3345)	5546 (4752–6922)	<0.001
Acoramidis, no. (%)	99 (62.7)	111 (70.3)	102 (64.6)	109 (69.0)	0.43
Age, y, mean±SD	75.66±6.79	76.90±6.88	78.01±5.81	78.52±6.35	<0.001
Sex, no. (%)					0.15
Male	141 (89.2)	137 (86.7)	149 (94.3)	143 (90.5)	
Female	17 (10.8)	21 (13.3)	9 (5.7)	15 (9.5)	
Race, no. (%)					0.77
White	139 (88.0)	140 (88.6)	137 (86.7)	139 (88.0)	
Black	8 (5.1)	5 (3.2)	9 (5.7)	8 (5.1)	
Asian	2 (1.3)	7 (4.4)	2 (1.3)	2 (1.3)	
Other*	9 (5.7)	6 (3.8)	10 (6.3)	9 (5.7)	
TTR genotype, no. (%)					0.92
Wild‐type	144 (91.1)	142 (89.9)	144 (91.1)	141 (89.2)	
Variant	14 (8.9)	16 (10.1)	14 (8.9)	17 (10.8)	
Genotype variant, no. (%)					0.29
V30M	1/13 (7.7)	0/16 (0.0)	0/14 (0.0)	0/15 (0.0)	
E89Q	0/13 (0.0)	1/16 (6.3)	0/14 (0.0)	0/15 (0.0)	
T60A	2/13 (15.4)	2/16 (12.5)	0/14 (0.0)	1/15 (6.7)	
V122I	6/13 (46.2)	6/16 (37.5)	11/14 (78.6)	13/15 (86.7)	
Other	4/13 (30.8)	7/16 (43.8)	3/14 (21.4)	1/15 (6.7)	
sTTR, mg/dL, median (Q1–Q3)	25 (22–28)	24 (20–27)	22 (19–26)	22 (18–26)	<0.001
eGFR, mL/min per 1.73 m^2^, mean±SD	69.11±19.72	64.42±15.95	61.58±15.15	48.73±15.68	<0.001
NYHA, no. (%)					<0.001
NYHA Class I	23 (14.6)	23 (14.6)	15 (9.5)	7 (4.4)	
NYHA Class II	119 (75.3)	116 (73.4)	108 (68.4)	112 (70.9)	
NYHA Class III	16 (10.1)	19 (12.0)	35 (22.2)	39 (24.7)	
KCCQ OSS, median (Q1–Q3)	81 (70–92)	73 (58–86)	70 (55–86)	67 (49–83)	<0.001
KCCQ CSS, median (Q1–Q3)	85 (72–95)	76 (64–89)	75 (62–88)	73 (54–85)	<0.001
KCCQ TSS, median (Q1–Q3)	88 (76–96)	82 (65–94)	80 (65–92)	73 (56–86)	<0.001
6MWD, meters, median (Q1–Q3)	394 (330–456)	372 (302–439)	353 (283–405)	306 (246–383)	<0.001
GNRI, median (Q1–Q3)	104 (103–107)	104 (103–107)	104 (101–107)	103 (101–106)	0.007

	Clinical characteristic by quartiles of baseline sTTR				*P* value
	Q1 (n=193)	Q2 (n=137)	Q3 (n=156)	Q4 (n=140)	
sTTR, mg/dL, median (Q1–Q3)	18 (15–19)	22 (21–23)	25 (24–26)	30 (28–33)	<0.001
Acoramidis, no. (%)	134 (69.4)	89 (65.0)	110 (70.5)	85 (60.7)	0.25
Age, y, mean±SD	77.78±6.60	77.10±6.90	77.98±6.10	75.94±6.56	0.03
Sex, no. (%)					0.67
Male	173 (89.6)	127 (92.7)	138 (88.5)	126 (90.0)	
Female	20 (10.4)	10 (7.3)	18 (11.5)	14 (10.0)	
Race, no. (%)					<0.001
White	157 (81.3)	122 (89.1)	142 (91.0)	129 (92.1)	
Black	23 (11.9)	3 (2.2)	3 (1.9)	0 (0.0)	
Asian	3 (1.6)	4 (2.9)	2 (1.3)	4 (2.9)	
Other^*^	10 (5.2)	8 (5.8)	9 (5.8)	7 (5.0)	
TTR Genotype, no. (%)					<0.001
Wild‐type	149 (77.2)	126 (92.0)	154 (98.7)	137 (97.9)	
Variant	44 (22.8)	11 (8.0)	2 (1.3)	3 (2.1)	
Genotype variant, no. (%)					<0.001
V30M	0/41 (0.0)	0/11 (0.0)	0/2 (0.0)	1/3 (33.3)	
E89Q	1/41 (2.4)	0/11 (0.0)	0/2 (0.0)	0 (0.0)	
T60A	2/41 (4.9)	3/11 (27.3)	0/2 (0.0)	0 (0.0)	
V122I	31/41 (75.6)	3/11 (27.3)	1/2 (50.0)	0 (0.0)	
Other	7/41 (17.1)	5/11 (45.5)	1/2 (50.0)	2/3 (66.7)	
NT‐proBNP, ng/L, median(Q1–Q3)	2786 (1803–4752)	2392 (1307–3692)	2038 (1074–3286)	1539 (1021–3061)	<0.001
eGFR, mL/min per 1.73 m^2^, mean±SD	61.76±18.34	64.54±18.57	62.07±18.45	55.47±16.51	<0.001
NYHA, no. (%)					0.26
NYHA Class I	13 (6.7)	20 (14.6)	18 (11.5)	17 (12.1)	
NYHA Class II	143 (74.1)	96 (70.1)	108 (69.2)	104 (74.3)	
NYHA Class III	37 (19.2)	21 (15.3)	30 (19.2)	19 (13.6)	
KCCQ OSS, median (Q1–Q3)	69 (52–83)	75 (60–88)	76 (63–87)	78 (62–91)	0.001
KCCQ CSS, median (Q1–Q3)	73 (59–86)	78 (62–91)	81 (67–90)	80 (67–93)	<0.001
KCCQ TSS, median (Q1–Q3)	75 (57–89)	82 (65–94)	85 (67–96)	83 (69–94)	0.001
6MWD, meters, median (Q1–Q3)	324 (246–398)	378 (304–438)	359 (293–430)	371 (317–441)	<0.001
GNRI, median (Q1–Q3)	103 (100–106)	104 (101–106)	106 (103–107)	106 (104–109)	<0.001

6MWD indicates 6‐minute walk distance; eGFR, estimated glomerular filtration rate; GNRI, Geriatric Nutritional Risk Index; KCCQ CSS, Kansas City Cardiomyopathy Questionnaire Clinical Summary Score; KCCQ OSS, Kansas City Cardiomyopathy Questionnaire Overall Symptom Score; KCCQ TSS, Kansas City Cardiomyopathy Questionnaire Total Symptom Score; NT‐proBNP, N‐terminal pro‐B‐type natriuretic peptide; NYHA, New York Heart Association; Q, quartile; and sTTR, serum transthyretin.

* Other includes American Indian or Alaska Native, Native Hawaiian or other Pacific Islander, multiple races, and not reported.

### Variables Associated With NT‐proBNP and sTTR

In bivariate correlation, a modest but statistically significant inverse correlation between NT‐proBNP and sTTR was present (rho −0.24; *P* < 0.001). In multivariable‐adjusted linear regression analyses, independent correlates of baseline NT‐proBNP included sex, TTR genotype, eGFR, and symptom severity as assessed by New York Heart Association class; additionally, baseline sTTR was a significant inverse correlate of NT‐proBNP (Table S1); GNRI results did not emerge as an independent variable associated with NT‐proBNP.

For sTTR, multivariable linear regression analyses indicated independent associations with age, TTR genotype, eGFR, and NT‐proBNP. In contrast to NT‐proBNP, GNRI emerged as an independent factor associated with sTTR concentrations; as expected, worse nutrition was independently associated with lower sTTR (Table S1).

### Prognostic Meaning of Biomarkers at Baseline

In univariable followed by multivariable Cox proportional hazards analyses and without inclusion of baseline sTTR concentrations, baseline NT‐proBNP concentrations were a significant predictor of ACM/first CVH through Month 30 (HR, 1.50 per SD increase in NT‐proBNP [95% CI, 1.29–1.73]; *P*<0.001) and CVM/first CVH through Month 30 (HR, 1.53 per SD increase in NT‐proBNP [95% CI, 1.31–1.78]; *P*<0.001; Table 2). In Kaplan–Meier analyses of baseline NT‐proBNP quartiles, a shorter time to first event for each outcome measure was noted with higher NT‐proBNP concentrations (all log rank *P*<0.001; Figure 1).

**Table 2:** Adjudicated Outcomes as a Function of Baseline Log2‐NT‐proBNP and Log2‐sTTR

Adjudicated outcomes as a function of baseline Log2‐NT‐proBNP						
Variable	Univariable			Multivariable		
	HR	95% CI	*P* value	HR	95% CI	*P* value
ACM/first CVH						
Baseline Log2‐transformed NT‐proBNP	1.67	1.46–1.90	<0.001	1.50	1.29–1.73	<0.001
Acoramidis treatment				0.63	0.49–0.81	<0.001
Baseline 6MWD				0.75	0.66–0.86	<0.001
Baseline eGFR				0.92	0.81–1.06	0.26
CVM/first CVH						
Baseline Log2‐transformed NT‐proBNP	1.66	1.45–1.91	<0.001	1.53	1.31–1.78	<0.001
Acoramidis treatment				0.59	0.46–0.77	<0.001
Baseline 6MWD				0.78	0.68–0.90	<0.001
Baseline eGFR				0.96	0.83–1.10	0.54

Adjudicated outcomes as a function of baseline Log2‐sTTR						
Variable	Univariable			Multivariable		
	HR	95% CI	*P* value	HR	95% CI	*P* value
ACM/first CVH						
Baseline Log2‐transformed sTTR	0.78	0.70–0.87	<0.001	0.78	0.70–0.88	<0.001
Acoramidis treatment				0.68	0.53–0.87	0.002
Baseline 6MWD				0.74	0.64–0.85	<0.001
Baseline eGFR				0.77	0.68–0.88	<0.001
CVM/first CVH						
Baseline Log2‐transformed sTTR	0.78	0.70–0.88	<0.001	0.79	0.70–0.89	<0.001
Acoramidis treatment				0.64	0.49–0.82	<0.001
Baseline 6MWD				0.76	0.66–0.88	<0.001
Baseline eGFR				0.80	0.69–0.91	<0.001

Outcomes were assessed using a Cox proportional hazards model. 6MWD indicates 6‐minute walk distance; ACM, all‐cause mortality; CVH, cardiovascular hospitalization; CVM, cardiovascular mortality; eGFR, estimated glomerular filtration rate; HR, hazard ratio; NT‐proBNP, N‐terminal pro–B‐type natriuretic peptide; and sTTR, serum transthyretin.

**Figure 1:**
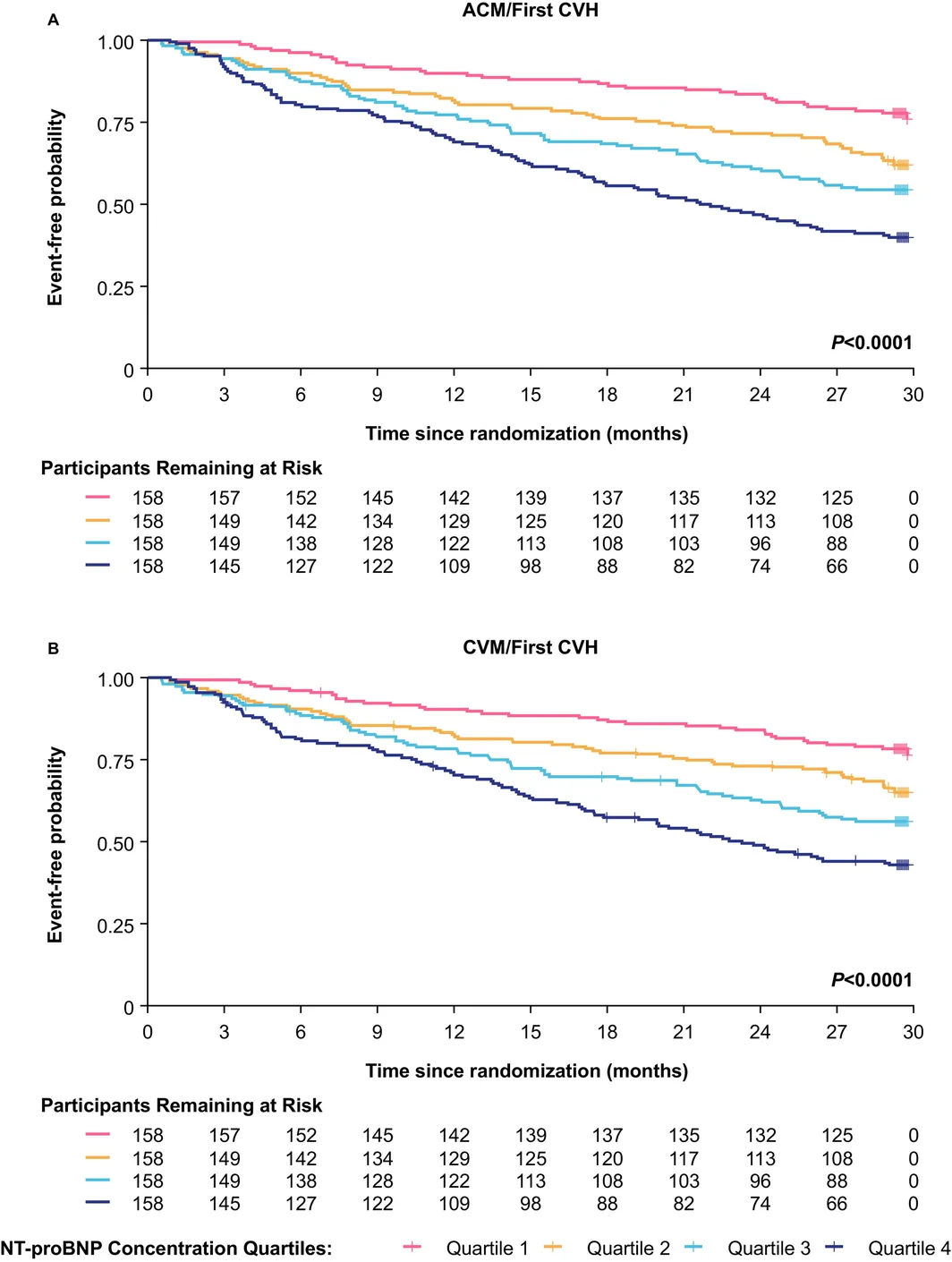
Kaplan–Meier curves detailing outcomes across baseline NT‐proBNP quartiles. Kaplan–Meier curves for (**A**) ACM/first CVH and (**B**) CVM/first CVH through Mo 30 stratified by baseline NT‐proBNP concentration quartiles. Quartile 1 purple, Quartile 2 dark yellow, Quartile 3 light blue, Quartile 4 dark blue – Quartile 1 represents the lowest NT‐proBNP concentration and Quartile 4 represents the highest. ACM indicates all‐cause mortality; CVH, cardiovascular hospitalization; CVM, cardiovascular mortality; and NT‐proBNP, N‐terminal pro‐B‐type natriuretic peptide.

In a similar manner, baseline sTTR concentrations were analyzed in multivariable Cox proportional hazard models; these models (performed without NT‐proBNP concentrations added) showed that higher sTTR concentrations were predictive of lower event rates. For example, baseline sTTR concentrations inversely predicted ACM/first CVH (HR, 0.78 per SD increase in sTTR [95% CI, 0.70–0.88]; *P*<0.001) and CVM/first CVH (HR, 0.79 per SD increase in sTTR [95% CI, 0.70–0.89]; *P*<0.001; Table 2).

To understand the potential impact of nutrition on the prognostic information provided by biomarker testing, GNRI scores were forced into the prognostic models (Table S2). In doing so, the HRs for NT‐proBNP and sTTR were unaffected, and GNRI was not a significant predictor.

In Kaplan–Meier analyses, lower baseline sTTR concentrations were generally associated with shorter time to first event, driven primarily by markedly worse outcomes in the lowest quartile (all log rank *P*<0.001; Figure 2).

**Figure 2:**
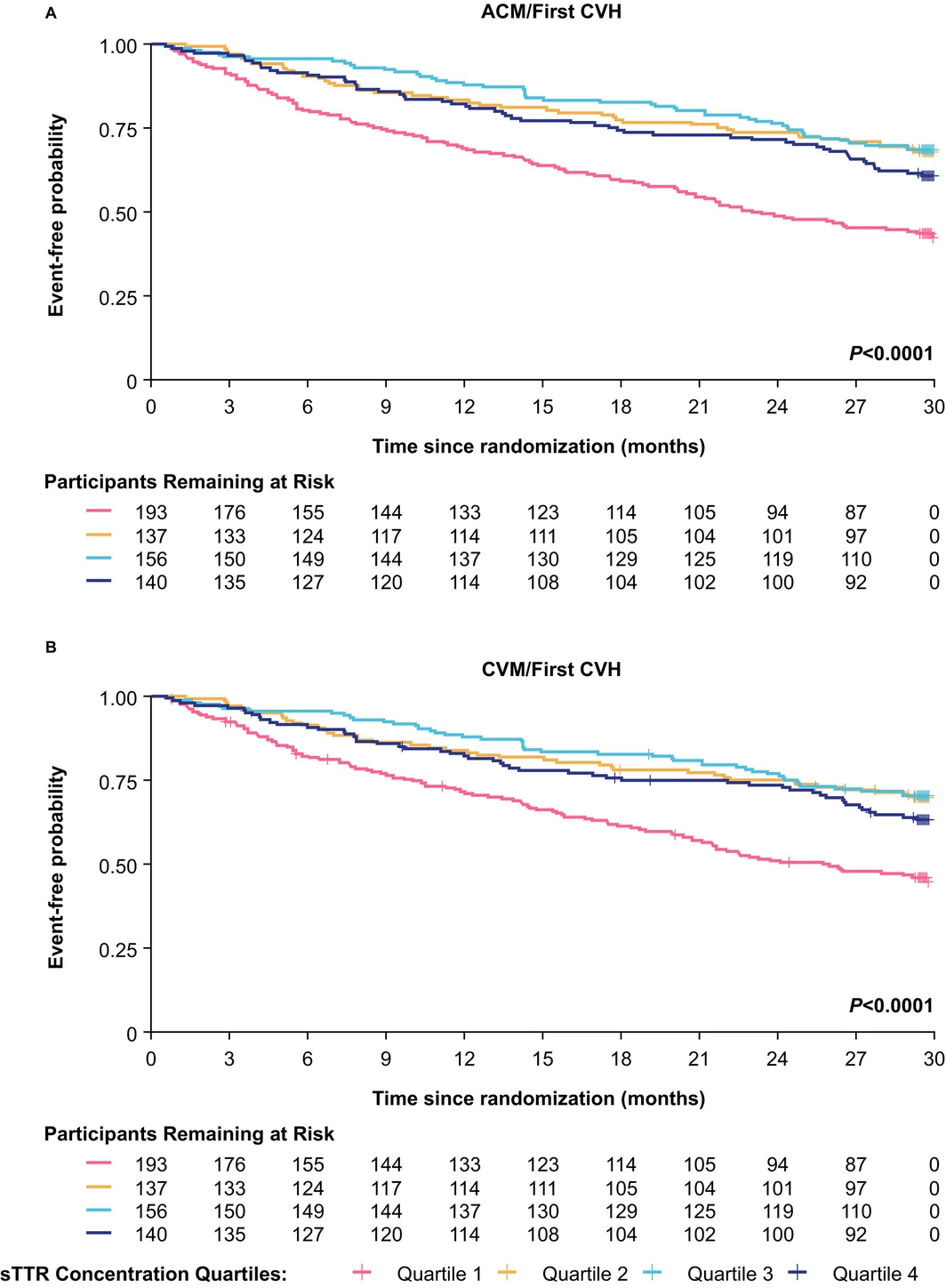
Kaplan–Meier curves detailing outcomes across baseline sTTR quartiles. Kaplan–Meier curves for (**A**) ACM/first CVH and (**B**) CVM/first cardiovascular hospitalization through Mo 30 stratified by baseline sTTR concentration quartiles. Quartile 1 purple, Quartile 2 dark yellow, Quartile 3 light blue, Quartile 4 dark blue—Quartile 1 represents the lowest sTTR concentration and Quartile 4 represents the highest. ACM indicates all‐cause mortality; CVH, cardiovascular hospitalization; CVM, cardiovascular mortality; and sTTR, serum transthyretin.

### Combination of Baseline sTTR and NT‐proBNP

Results for baseline NT‐proBNP and sTTR in relation to KCCQ overall summary score and 6MWD are detailed in Table 3. In this analysis, study participants with NT‐proBNP ≥median (2326 ng/L) and sTTR <median (23 mg/dL) at baseline also had the worst health status (reflected in lower KCCQ overall summary score, clinical summary score, and total summary score) and lowest 6MWD. Clinical outcomes were then evaluated using both biomarkers.

**Table 3:** Baseline KCCQ Scores and 6MWD Categorized by NT‐proBNP and sTTR Medians

	Low NT‐proBNP High sTTR (n=204)	Low NT‐proBNP Low sTTR (n=110)	High NT‐proBNP High sTTR (n=144)	High NT‐proBNP Low sTTR (n=168)	*P* value
KCCQ Overall Summary Score, median (Q1–Q3)	80 (66–90)	74 (63–88)	74 (56–87)	66 (51–83)	<0.001
KCCQ Clinical Summary Score, median (Q1–Q3)	83 (71–94)	77 (67–89)	79 (61–90)	72 (54–83)	<0.001
KCCQ Total Summary Score, median (Q1–Q3)	85 (71–96)	83 (66–96)	81 (63–92)	73 (56–86)	<0.0001
6MWD, median (Q1–Q3)	391 (329–452)	365 (289–441)	351 (295–406)	313 (238–388)	<0.001

Low NT‐proBNP concentration was defined as being below the median baseline value (ie, <2326 ng/L); high NT‐proBNP concentration was defined as above or equal to the median baseline value (ie, ≥2326 ng/L). Low sTTR concentration was defined as being below the median baseline value (ie, <23 mg/dL); high sTTR concentration was defined as being at or above the median baseline value (ie, ≥23 mg/dL). 6MWD indicates 6‐minute walk distance; KCCQ, Kansas City Cardiomyopathy Questionnaire; NT‐proBNP, N‐terminal pro‐B‐type natriuretic peptide; Q, quartile; and sTTR, serum transthyretin.

In multivariable Cox models that included both baseline NT‐proBNP and sTTR and were adjusted for treatment assignment, 6MWD, and eGFR, both biomarkers remained statistically significant predictors of all outcomes examined (Table 4). As with the individual biomarkers, forced addition of GNRI to the prognostic model did not attenuate the prognostic value of NT‐proBNP and sTTR (Table S2). Similar findings were observed when median‐based biomarker categorization was evaluated (Table S3).

**Table 4:** Joint Cox Proportional Hazards Modeling Including Both Baseline Log2‐NT‐proBNP and Log2‐sTTR

Variable	Univariable			Multivariable		
	HR	95% CI	*P* value	HR	95% CI	*P* value
ACM/first CVH						
Baseline Log2‐transformed NT‐proBNP	1.59	1.39–1.83	<0.001	1.41	1.21–1.64	<0.001
Baseline Log2‐transformed sTTR	0.84	0.75–0.94	0.003	0.84	0.74–0.95	0.006
Acoramidis treatment				0.64	0.50–0.82	<0.001
Baseline 6MWD				0.77	0.67–0.89	<0.001
Baseline eGFR				0.88	0.76–1.01	0.08
CVM/first CVH						
Baseline Log2‐transformed NT‐proBNP	1.59	1.38–1.83	<0.001	1.44	1.23–1.69	<0.001
Baseline Log2‐transformed sTTR	0.85	0.75–0.95	0.006	0.85	0.75–0.97	0.01
Acoramidis treatment				0.60	0.46–0.78	<0.001
Baseline 6MWD				0.81	0.70–0.93	0.003
Baseline eGFR				0.91	0.79–1.06	0.23

6MWD indicates 6‐minute walk distance; ACM, all‐cause mortality; CVH, cardiovascular hospitalization; CVM, cardiovascular mortality; eGFR, estimated glomerular filtration rate; HR, hazard ratio; NT‐proBNP, N‐terminal pro‐B‐type natriuretic peptide; and sTTR, serum transthyretin.

In C‐statistic analyses, adding sTTR to NT‐proBNP resulted in an increase in C‐statistic or improvement in calibration (as assessed using AIC and BIC): using continuous log2‐standardized results, the C‐statistic increased, whereas the AIC and BIC were minimized; using median cuts for both variables, calibration improved. A model including NT‐proBNP/sTTR cut at their medians had a base C‐statistic of 0.64; addition of NAC staging did not change the C‐statistic and resulted in worse model calibration with an increase in both AIC (from 3141.5 to 3145.1) and BIC (from 3148.7 to 3159.4).

Study participants were then categorized as a function of their baseline NT‐proBNP and sTTR. Event rates by 30 months were consistently highest in those with NT‐proBNP ≥median (2326 ng/L) and sTTR<median (23 mg/dL) at baseline, with a higher risk for each adjudicated cardiovascular outcome noted (Figure 3); event rates per 100 person‐years are also shown in Figure 3. Further, Kaplan–Meier analyses jointly considering NT‐proBNP and sTTR, divided using baseline median concentrations revealed that those with higher baseline NT‐proBNP and lower sTTR at baseline had considerably shorter time to ACM/first CVH or CVM/first CVH (all log rank *P*<0.001; Figure 4).

**Figure 3:**
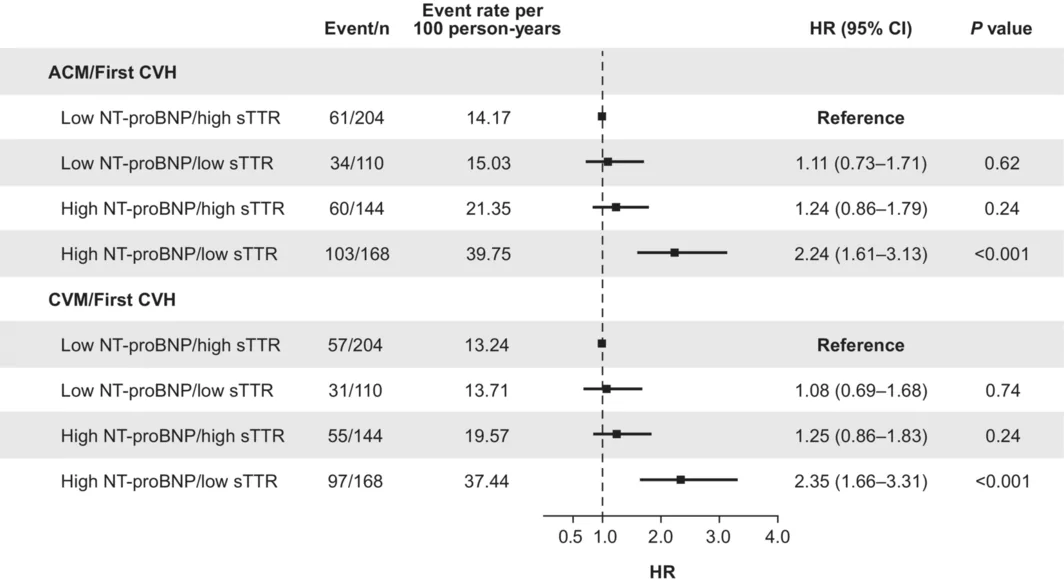
Forest plot for outcomes by baseline NT‐proBNP and sTTR concentrations. Outcomes are shown for subgroups stratified by NT‐proBNP and sTTR concentrations. Low NT‐proBNP concentration was defined as being below the median baseline value (ie, <2326 ng/L); high NT‐proBNP concentration was defined as above or equal to the median baseline value (ie, ≥2326 ng/L). Low sTTR concentration was defined as being below the median baseline value (ie, <23 mg/dL); high sTTR concentration was defined as being at or above the median baseline value (ie, ≥23 mg/dL). ACM indicates all‐cause mortality; CVH, cardiovascular hospitalization; CVM, cardiovascular mortality; HR, hazard ratio; NT‐proBNP, N‐terminal pro‐B‐type natriuretic peptide; and sTTR, serum transthyretin.

**Figure 4:**
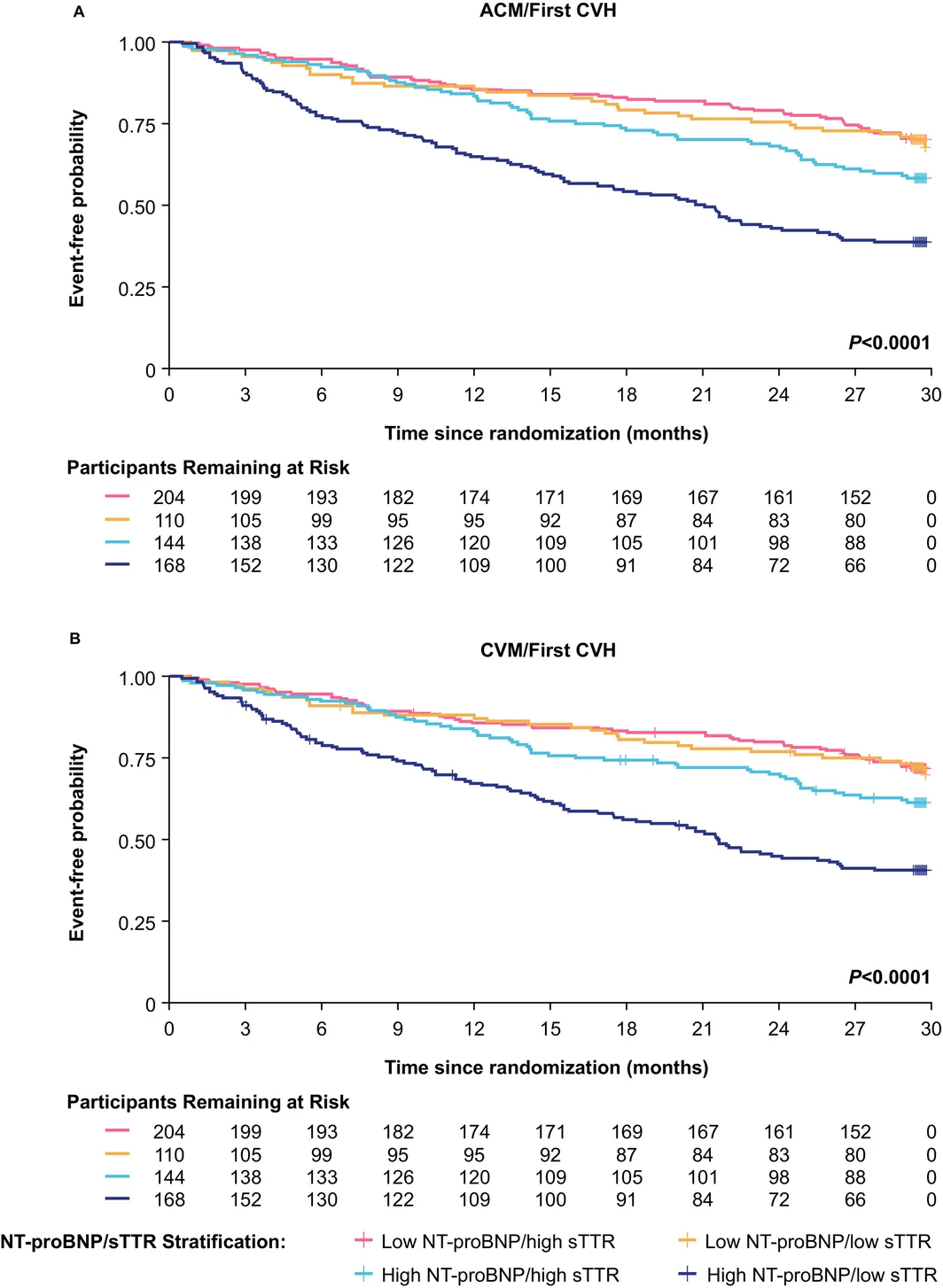
Kaplan–Meier curves for outcomes by baseline NT‐proBNP and sTTR concentrations. Kaplan–Meier curves for (**A**) ACM/first CVH and (**B**) CVM/first CVH through Mo 30. Low NT‐proBNP concentration was defined as being below the median baseline value (ie, <2326 ng/L); high NT‐proBNP concentration was defined as above or equal to the median baseline value (ie, ≥2326 ng/L). Low sTTR concentration was defined as being below the median baseline value (ie, <23 mg/dL); high sTTR concentration was defined as being at or above the median baseline value (ie, ≥23 mg/dL). ACM indicates all‐cause mortality; CVH, cardiovascular hospitalization; CVM, cardiovascular mortality; NT‐proBNP, N‐terminal pro‐B‐type natriuretic peptide; and sTTR, serum transthyretin.

### Effect of Acoramidis

Across baseline biomarker categories, treatment with acoramidis reduced adjudicated ACM/first CVH and CVM/first CVH event rates (Figure 5) without heterogeneity (all interaction *P* values were >0.05). Interaction testing for heterogeneity of prognostic results within GNRI values was similarly negative (*P*=0.40).

**Figure 5:**
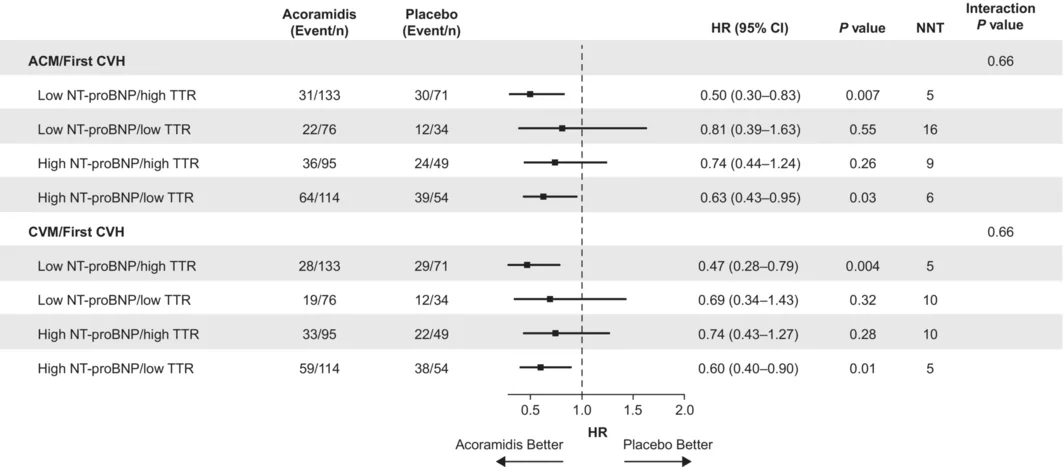
Forest plot for outcomes by treatment, NT‐proBNP, and sTTR. Outcomes are shown for the interaction between treatment and baseline NT‐proBNP and sTTR concentrations. Low NT‐proBNP concentration was defined as being below the median baseline value (ie, <2326 ng/L); high NT‐proBNP concentration was defined as above or equal to the median baseline value (ie, ≥2326 ng/L). Low sTTR concentration was defined as being below the median baseline value (ie, <23 mg/dL); high sTTR concentration was defined as being at or above the median baseline value (ie, ≥23 mg/dL). ACM indicates all‐cause mortality; CVH, cardiovascular hospitalization; CVM, cardiovascular mortality; HR, hazard ratio; NNT, number needed to treat; NT‐proBNP, N‐terminal pro‐B‐type natriuretic peptide; and sTTR, serum transthyretin.

Participants with elevated NT‐proBNP and low sTTR at baseline were at highest risk for ACM/first CVH and CVM/first CVH; however, the risk reduction from acoramidis treatment in this high‐risk group was consistent with that observed in lower‐risk groups (Figure 5). The numbers needed to treat to reduce any ACM/first CVH or CVM/first CVH events were generally consistent across all combinations of NT‐proBNP/sTTR risk groups (5, 16, 9, and 6, and 5, 10, 10, and 5, respectively, for participants with low NT‐proBNP/high sTTR, low NT‐proBNP/low sTTR, high NT‐proBNP/high sTTR, and high NT‐proBNP/low sTTR; *P* value=nonsignificant for interaction). Notably, numbers needed to treat were also generally consistent across NAC stages (17, 12, 13, and 14, 10 and 11, respectively, for participants with NAC stages I, II and III; *P* value=nonsignificant for interaction).

## DISCUSSION

In this post hoc analysis of baseline biomarker data from the ATTRIBUTE‐CM trial, there are 5 major findings: (1) an independent inverse correlation was observed between NT‐proBNP and sTTR; (2) higher NT‐proBNP and lower sTTR concentrations were associated with lower KCCQ scores and shorter 6MWD at baseline; (3) when separately evaluated in multivariable models, higher NT‐proBNP and lower sTTR at baseline were significantly correlated with time to ACM/first CVH and CVM/first CVH through Month 30, (4) in joint multivariable models, higher baseline NT‐proBNP and lower sTTR concentrations were each significantly associated with worse HF related health status (as assessed by KCCQ overall summary score), lowest functional capacity (as assessed by 6MWD), and worse subsequent clinical outcomes with considerably shorter time to first cardiovascular event, including death; and (5) acoramidis demonstrated consistent clinical efficacy across all subgroups categorized by NT‐proBNP and sTTR regardless of baseline biomarker concentrations. Although nutritional status (evaluated using GNRI) was an independent variable associated with sTTR concentrations, the prognostic meaning of sTTR was independent of the GNRI. These data extend the current understanding of biomarker testing in ATTR‐CM and create the novel, important concept of joint measurement of NT‐proBNP and sTTR to better understand prognosis in patients with ATTR‐CM.

The inverse relationship between these biomarkers is noteworthy: findings from this study suggest that lower sTTR is associated with higher NT‐proBNP in fully adjusted models, and in turn, both are associated with worse outcomes. NT‐proBNP and sTTR likely reflect distinct yet complementary and independently informative aspects of the ATTR‐CM disease process. Baseline, untreated circulating sTTR concentration primarily reflects the degree of TTR stabilization and, indirectly, the extent of ongoing tetramer dissociation and generation of toxic amyloidogenic precursors.^16^, ^17^ In contrast, NT‐proBNP reflects myocardial wall stress and dysfunction that occur downstream as a result of amyloid fibril deposition.^10^, ^12^, ^19^ We speculate that the presence and degree of TTR destabilization may mediate cardiomyocyte stress and injury resulting in greater NT‐proBNP release.^2^ Together, they may serve as complementary indicators along the pathophysiologic continuum of ATTR‐CM.

In the landmark ATTRIBUTE‐CM trial report, treatment with acoramidis blunted the increase in NT‐proBNP observed with placebo and was associated with a reduction from baseline in NT‐proBNP in almost half of participants at Month 30.^15^, ^20^ Acoramidis also led to an early increase in sTTR following initiation of treatment, which was favorably prognostic,^15^, ^17^ but its relationship to other prognostic biomarkers such as NT‐proBNP remained to be fully elucidated; our study adds to this body of knowledge. In the present analysis, baseline concentrations of both NT‐proBNP and sTTR were prognostic for key adjudicated end points in the trial, including mortality. When considered in joint models, each remained prognostically important: higher NT‐proBNP and lower sTTR at baseline had a considerably higher risk for adverse outcomes, with a tripling of event rates compared with those with lower NT‐proBNP and higher sTTR. These biomarker patterns were also significantly associated with baseline KCCQ and 6MWD. It is reassuring to note that acoramidis reduced risk for time to ACM/first CVH and CVM/first CVH regardless of baseline biomarker status with a generally consistent number needed to treat to prevent one event by the 30‐month follow‐up.

A question arises as to whether malnutrition—an important comorbidity in HF syndromes including ATTR‐CM—might mediate the prognostic information from sTTR measurement.^21^, ^22^, ^23^, ^24^ The results from the current analysis suggest that in the context of this generally well‐nourished clinical trial population, lower GNRI scores were associated with lower sTTR. Despite this, in prognostic models adjusted for nutritional status, lower sTTR remained an independent predictor of outcomes. These data suggest that although nutrition plays a general role in determining sTTR concentrations, other factors leading to lower sTTR, such as decreased circulating tetramer owing to destabilization, misfolding into monomers, and subsequent incorporation into amyloid fibrils, may play a more important role.

This analysis highlights the potential to further improve risk stratification using circulating biomarkers. Our data extend the value of NT‐proBNP and add sTTR measurement as an important disease marker and indicator of adverse outcomes in ATTR‐CM. Given the objective nature of biomarkers and their ease of use, low cost, and wide availability, measurement of NT‐proBNP and sTTR could inform future efforts to enhance existing ATTR‐CM staging systems. Combining sTTR and NT‐proBNP—a highly validated marker of myocardial stress and neurohormonal activation—would augment other known markers of disease progression such as clinical/functional end points and imaging biomarkers.^11^, ^25^ Notably, comparing NT‐proBNP/sTTR to the NAC stage suggested that the former provided prognostic information at least comparable to the NAC stage; since the NAC stage includes NT‐proBNP, this implies the addition of eGFR to the model did not further discriminate outcomes beyond NT‐proBNP/sTTR.

### Study Limitations

Although the ATTRIBUTE‐CM trial enrolled a contemporary cohort of patients with ATTR‐CM, it may not fully represent all patients with ATTR‐CM seen in clinics due to the rapidly evolving disease landscape. This post hoc analysis included only participants for whom sTTR concentrations were available at baseline; missing data were negligible (n=6). The lack of definitive upper reference limits for sTTR and NT‐proBNP for use in ATTR‐CM required continuous modeling of these variables and the use of medians for the trial population; use of log2‐transformed data ensures a robust estimation of the full range of marker concentrations, whereas medians are less influenced by extreme outliers or values near the limits of assay detection, providing a more stable baseline for exploratory risk stratification. It is reassuring the NT‐proBNP median in this population approximates that of the NAC threshold of 3000 ng/L. More data are needed regarding the combined measurement of NT‐proBNP and sTTR in those treated with stabilizers, particularly following initiation of therapy; although early sTTR increases after acoramidis treatment is associated with better outcomes, it remains unclear whether secular trends in sTTR and NT‐proBNP are useful for prognostic monitoring of stabilizer therapy; such analyses bear exploration. ATTRIBUTE‐CM included low proportions of women, participants who identified as Black, and participants with variant ATTR‐CM. Recent studies have reported underdiagnosis of ATTR‐CM in women^26^ and in Black individuals,^27^ and variant ATTR‐CM is known to have a generally worse prognosis than wild‐type ATTR‐CM.^28^, ^29^ This is very relevant as both race and variant genotype were associated with lower sTTR concentration; more data are needed in these underrepresented populations. Although the GNRI is validated among individuals with HF, the GNRI can underestimate malnutrition because edema, fluid overload, and increased body weight from congestion or sarcopenic obesity can falsely elevate the weight component, making patients look “better nourished” than they are. To our knowledge, this is the first analysis of the role of the GNRI in those with ATTR‐CM; more data about the GNRI are needed in people affected by this disease. Study participants in the ATTRIBUTE‐CM trial were not permitted to be treated with open‐label, commercial tafamidis for the first 12 months of the trial, and otherwise initiated tafamidis as a concomitant medication at random with respect to timing of initiation and duration of concomitant therapy, with only a minority of patients (~20%) being exposed to concomitant tafamidis treatment during the study. No participants received TTR knock‐down therapies (small interfering RNA or antisense oligonucleotides) at any time during the study. How the data in this analysis would apply in populations treated with these agents remains unclear; at least with respect to knockdown agents, the prognostic meaning of TTR would be expected to differ from those not treated with such therapies. Both body mass index and kidney function are associated with concentrations of NT‐proBNP; in fully adjusted models, neither factored into the link between lower sTTR and higher NT‐proBNP, however. Lastly, although compelling, the biomarker data in this post hoc analysis cannot yet be leveraged to replace widely used risk models such as the NAC risk model.

## CONCLUSIONS

These results, based on baseline and outcomes data from ATTRIBUTE‐CM, extend the understanding of biomarkers in individuals with ATTR‐CM. This analysis suggests that NT‐proBNP and sTTR reflect distinct yet complementary aspects of the disease process and that their joint evaluation may enhance risk stratification for patients with ATTR‐CM. Participants with higher NT‐proBNP and lower sTTR at baseline had the highest rates of ACM/CVH or CVM/CVH. Acoramidis demonstrated consistent clinical efficacy regardless of baseline biomarker concentrations. Further research is warranted to better define the role of biomarkers in this high‐risk population.

## Disclosures

Onyedika John Ilonze has received consultancy fees from AstraZeneca and BridgeBio Pharma.He receives grant support from the American Heart Association Career Development Award (CDA) 26CDA1591379, Robert A. Winn Career Development Award and the Krannert Cardiovascular Research Center, Family Medicine, Clinical Pharmacology, and HEALER. These awards are unrelated to the current work. Daniel Philip Judge has received consultancy fees from Alexion Pharmaceuticals, Alleviant Medical, Alnylam Pharmaceuticals, Attralus, BridgeBio Pharma, Cytokinetics, Lexeo Therapeutics, Novo Nordisk, Pfizer, Renovacor, Rocket Pharmaceuticals, and Tenaya Therapeutics; and his institution received clinical trial funding from Array Biopharma, BridgeBio Pharma, Inc., MyoKardia, and Pfizer. Ahmad Masri has acted as a researcher for Attralus, Cytokinetics, Ionis Pharmaceuticals, and Pfizer; and as a consultant, advisor, or speaker for Akros Pharma, Alexion Pharmaceuticals, Alnylam Pharmaceuticals, AstraZeneca, Attralus, BioMarin Pharmaceutical, BridgeBio Pharma, Inc., Bristol Myers Squibb, Cytokinetics, Haya Therapeutics, Ionis Pharmaceuticals, Lexicon Pharmaceuticals, Pfizer, Prothena Biosciences, and Tenaya Therapeutics. Prem Soman has received grants from Pfizer and acted as a consultant, advisor, or speaker for BridgeBio Pharma, Inc., Alnylam, Pfizer, and Spectrum Dynamics. Francesco Cappelli has acted as a consultant, advisor, or speaker for Alnylam Pharmaceuticals, AstraZeneca, Bayer, BridgeBio Pharma, Bristol Myers Squibb, Novo Nordisk, and Pfizer. Michel Georges Khouri has acted as a researcher for Alnylam Pharmaceuticals, AstraZeneca, Attralus, BridgeBio Pharma, Ionis Pharmaceuticals, and Pfizer; and as a consultant, advisor, or speaker for Alnylam Pharmaceuticals, AstraZeneca, BridgeBio Pharma, and Pfizer. Nitasha Sarswat has received research funding from Pfizer; her institution has received funding for clinical trials from Alnylam Pharmaceuticals, AstraZeneca, BridgeBio Pharma, Intellia Therapeutics, Novo Nordisk, and Pfizer; and her institution has received advisor funding for Alnylam Pharmaceuticals, AstraZeneca, BridgeBio Pharma, Novo Nordisk, and Pfizer. Mazen Hanna has served on advisory boards for Alexion Pharmaceuticals, Alnylam Pharmaceuticals, BridgeBio Pharma, Ionis Pharmaceuticals, Attralus, Novo Nordisk, and Pfizer. Yuxi Liu conducted statistical analyses for BAIM Institute, which received financial support from BridgeBio Pharma, Inc., in connection with this study/article. Gillian Murtagh, Kuangnan Xiong, Adam Castaño, Jean‐François Tamby, Jonathan C. Fox, and Uma Sinha are employees and stakeholders of BridgeBio Pharma, Inc. Jan Marie Griffin has acted as a consultant for AstraZeneca, BridgeBio Pharma, Inc., and Pfizer; and has received research grants from BridgeBio Pharma, Inc. and Pfizer. Julian D. Gillmore has acted as a consultant, advisor, or speaker for Alnylam Pharmaceuticals, AstraZeneca, Attralus, BridgeBio Pharma, Inc., Intellia Therapeutics, Ionis Pharmaceuticals, Lycia Therapeutics, and Pfizer; and has received institutional research grants from Alnylam Pharmaceuticals and AstraZeneca. James Louis Januzzi has received grants from Abbott Diagnostics, Applied Therapeutics, AstraZeneca, BridgeBio Pharma, BMS, and Novartis; has acted as a consultant, advisor, or speaker for Abbott Diagnostics, Beckman‐Coulter, Jana Care, Janssen, Novartis, Prevencio, Quidel, and Roche Diagnostics; has served on clinical end point committees/data safety monitoring boards for Abbott, AbbVie, Amgen, CVRx, Medtronic, Pfizer, and Roche Diagnostics; and has equity in Fibrosys, Imbria Pharma, Jana Care, and Prevencio.

## Source of Funding

The ATTRibute‐CM study was funded by BridgeBio Pharma, Inc., San Francisco, CA, USA. The statistical support for the analyses included in this article was conducted by BAIM Institute for Clinical Research and funded by BridgeBio Pharma, Inc. San Francisco, CA.

## Role of Funder/Sponsor

BridgeBio Pharma, Inc., designed and funded the ATTRIBUTE‐CM and its open‐label extension study and led all aspects of the study design, data collection, and study management. In their role as authors, employees of BridgeBio Pharma, Inc., contributed to the analysis and interpretation of the data, and participated in reviewing, and approving the article for submission. Additionally, BridgeBio Pharma, Inc., also reviewed the article for scientific and medical accuracy, approving it from an intellectual property perspective, and ensuring adherence with Good Publication Practice guidelines and International Committee of Medical Journal Editors recommendations, but had no right to veto the publication or submission of this article. The interpretation of the data and the decision to submit the article to the journal were completed independently by the authors without any influence from the sponsor of the study.

## Supporting information

CONSORT 2025 Checklist

Tables S1–S3
