# Supplementary material for: Natriuretic Peptide and Transthyretin and Their Relationship With Clinical Outcomes: Results From ATTRIBUTE‐CM, a Randomized Controlled Trial
Source: J Am Heart Assoc. 2026 Aug 30;15(19):e050566. doi: 10.1161/JAHA.126.050566 (PMC13745106; doi:10.1161/JAHA.126.050566)
Supplement: Supplementary file 1 [file JAH3-15-e050566-s001.pdf]

Supplemental Material

Table S1: Univariable and Multivariable Linear Regression Analysis for Correlates to Baseline Log2-NT-proBNP and Log2-sTTR

	Linear Regression Analysis for Correlates to Baseline Log2-NT-proBNP			
	**Univariable**		**Multivariable**	
	**Coefficient**	***P* value**	**Coefficient**	***P* value**
Age	0.03	<0.001	0.01	0.10
Sex, Male	0.12	0.44	0.37	0.006
Race, Ref = Other				
White	−0.04	0.86		
Black	−0.09	0.76		
Asian	−0.23	0.54		
TTR Genotype, Wild-type	0.07	0.65	0.30	0.04
eGFR	−0.03	<0.001	−0.03	<0.001
NYHA, Ref = Class I				
NYHA CLASS II	0.39	0.008	0.11	0.41
NYHA CLASS III	0.88	<0.001	0.39	0.01
Log2-sTTR	−0.69	<0.001	−0.92	<0.001
GNRI	−0.04	<0.001	0.003	0.74
	Linear Regression Analysis for Correlates to Baseline Log2-sTTR			
	**Univariable**		**Multivariable**	

	**Coefficient**	***P* value**	**Coefficient**	***P* value**
Age	−0.002	0.41	−0.006	0.008
Sex, Male	−0.001	0.99		
Race, Ref = Other				
White	0.07	0.29		
Black	−0.52	<0.001		
Asian	0.05	0.65		
TTR Genotype, Wild-type	0.45	<0.001	0.47	<0.001
eGFR	−0.003	0.002	−0.006	<0.001
NYHA, Ref = Class I				
NYHA CLASS II	−0.10	0.05		
NYHA CLASS III	−0.11	0.06		
Log2-NT-proBNP	−0.08	<0.001	−0.11	<0.001
GNRI	0.04	<0.001	0.03	<0.001

Variables in the multivariable model were selected using LASSO. Both ATTR-CM wild-type and variant participants were included in the model; “variant” served as the reference category for genotype.

ACM indicates all-cause mortality; ATTR-CM, transthyretin amyloid cardiomyopathy; CVH, cardiovascular hospitalization; CVM, cardiovascular mortality; eGFR, estimated glomerular filtration rate; GNRI, Geriatric Nutritional Risk Index; LASSO, Least Absolute Shrinkage and Selection Operator; NYHA, New York Heart Association; and sTTR, serum transthyretin.

Table S2: Joint Cox proportional hazards modeling with GNRI forced into the model for NT-proBNP alone, sTTR alone, and the combination of NT-proBNP/sTTR

**Joint Cox proportional hazards modeling with GNRI forced into the model for NT-proBNP alone**						
**ACM/First CVH**						
	**Univariable**			**Multivariable**		
**Variable**	**HR**	**95% CI**	***P* value**	**HR**	**95% CI**	***P* value**
Baseline Log2-transformed NT-proBNP	1.67	1.46–1.90	<0.001	1.49	1.28–1.73	<0.001
Acoramidis treatment				0.64	0.50–0.82	<0.001
Baseline 6MWD				0.76	0.66–0.87	<0.001
Baseline eGFR				0.93	0.81–1.06	0.27
GNRI				0.97	0.86–1.11	0.67
**Joint Cox proportional hazards modeling with GNRI forced into the model for sTTR alone**						
**ACM/First CVH**						
	**Univariable**			**Multivariable**		
**Variable**	**HR**	**95% CI**	***P* value**	**HR**	**95% CI**	***P* value**
Baseline Log2-transformed sTTR	0.78	0.70–0.87	<0.001	0.77	0.67–0.89	<0.001

Acoramidis treatment				0.68	0.52–0.87	0.002
Baseline 6MWD				0.74	0.65–0.85	<0.001
Baseline eGFR				0.77	0.68–0.88	<0.001
GNRI				1.03	0.89–1.18	0.72
**Joint Cox proportional hazards modeling with GNRI forced into the model for the combination of NT-proBNP/sTTR**						
	**Univariable**			**Multivariable**		
**Variable**	**HR**	**95% CI**	***P* value**	**HR**	**95% CI**	***P* value**
Baseline Log2-transformed NT-proBNP	1.59	1.39–1.83	<0.001	1.41	1.21–1.65	<0.001
Baseline Log2-transformed sTTR	0.84	0.75–0.94	0.003	0.84	0.72–0.97	0.02
Acoramidis treatment				0.64	0.50–0.82	<0.001
Baseline 6MWD				0.78	0.67–0.89	<0.001
Baseline eGFR				0.88	0.76–1.02	0.08
GNRI				1.03	0.90–1.18	0.68

6MWD indicates 6-minute walk distance; CI, confidence interval; eGFR, estimated glomerular filtration rate; GNRI, Geriatric Nutritional Risk Index; HR, hazard ratio; NT-proBNP, N-terminal pro–B-type natriuretic peptide; and sTTR, serum transthyretin.

Table S3: Joint Cox proportional hazards modeling with GNRI forced into the model, divided by the median split for NT-proBNP alone, sTTR alone, and the combination of NT-proBNP/sTTR

**Joint Cox proportional hazards modeling with GNRI forced into the model for NT-proBNP alone**						
**ACM/First CVH**						
	**Univariable**			**Multivariable**		
**Variable**	**HR**	**95% CI**	***P* value**	**HR**	**95% CI**	***P* value**
Baseline NT-proBNP at/above median	2.11	1.64–2.71	<0.001	1.65	1.26–2.16	<0.001
Acoramidis treatment				0.68	0.53–0.88	0.003
Baseline 6MWD				0.73	0.64–0.84	<0.001
Baseline eGFR				0.87	0.76–0.99	0.03
GNRI				0.96	0.85–1.09	0.60
**Joint Cox proportional hazards modeling with GNRI forced into the model for sTTR alone**						
**ACM/First CVH**						
	**Univariable**			**Multivariable**		
**Variable**	**HR**	**95% CI**	***P* value**	**HR**	**95% CI**	***P* value**
Baseline sTTR at/above median	0.60	0.47–0.77	<0.001	0.61	0.47–0.80	<0.001

Acoramidis treatment				0.68	0.52–0.87	0.002
Baseline 6MWD				0.74	0.64–0.85	<0.001
Baseline eGFR				0.78	0.69–0.89	<0.001
GNRI				1.01	0.88–1.15	0.94
**Joint Cox proportional hazards modeling with GNRI forced into the model for the combination of NT-proBNP/sTTR**						
	**Univariable**			**Multivariable**		
**Variable**	**HR**	**95% CI**	***P* value**	**HR**	**95% CI**	***P* value**
Baseline NT-proBNP at/above median	1.97	1.53–2.55	<0.001	1.54	1.17–2.03	0.002
Baseline sTTR at/above median	0.66	0.52–0.85	<0.001	0.65	0.49–0.85	0.001
Acoramidis treatment				0.67	0.52–0.86	0.002
Baseline 6MWD				0.76	0.66–0.87	<0.001
Baseline eGFR				0.83	0.73–0.95	0.008
GNRI				1.02	0.89–1.17	0.70

6MWD indicates 6-minute walk distance; CI, confidence interval; eGFR, estimated glomerular filtration rate; GNRI, Geriatric Nutritional Risk Index; HR, hazard ratio; NT-proBNP, N-terminal pro–B-type natriuretic peptide; and sTTR, serum transthyretin.
